# Supplementary material for: Latent-TGF-β has a domain swapped architecture
Source: Res Sq. 2024 Nov 1:rs.3.rs-5154292. Preprint. [Version 1] doi: 10.21203/rs.3.rs-5154292/v1 (PMC11581116; doi:10.21203/rs.3.rs-5154292/v1)
Supplement: Supplement 1 [file NIHPPRS5154292V1-supplement-1.pdf]

Supplementary Files

This is a list of supplementary files associated with this preprint. Click to download.

- FigS1.jpg
